# Design of ultra-thin underwater acoustic metasurface for broadband low-frequency diffuse reflection by deep neural networks

**DOI:** 10.1038/s41598-022-16312-1

**Published:** 2022-07-14

**Authors:** Ruichen Li, Yutong Jiang, Rongrong Zhu, Yijun Zou, Lian Shen, Bin Zheng

**Affiliations:** 1 Interdisciplinary Center for Quantum Information, State Key Laboratory of Modern Optical Instrumentation, ZJU-Hangzhou Global Scientific and Technological Innovation Center, Zhejiang University, Hangzhou, 310027 China; 2 School of Information and Electrical Engineering, Zhejiang University City College, Zhejiang, 310015 China; 3 International Joint Innovation Center, Key Lab. of Advanced Micro/Nano Electronic Devices & Smart Systems of Zhejiang, The Electromagnetics Academy at Zhejiang University, Zhejiang University, Haining, 314400 China; 4 Jinhua Institute of Zhejiang University, Zhejiang University, Jinhua, 321099 China

**Keywords:** Materials science, Physics

## Abstract

Underwater acoustic metasurfaces have broad application prospects for the stealth of underwater objects. However, problems such as a narrow operating frequency band, poor operating performance, and considerable thickness at low frequencies remain. In this study a reverse design method for ultra-thin underwater acoustic metasurfaces for low-frequency broadband is proposed using a tandem fully connected deep neural network. The tandem neural network consists of a pre-trained forward neural network and a reverse neural network, based on which a set of elements with flat phase variation and an almost equal phase shift interval in the range of 700–1150 Hz is designed. A diffuse underwater acoustic metasurface with 60 elements was designed, showing that the energy loss of the metasurface in the echo direction was greater than 10 dB. Our work opens a novel pathway for realising low-frequency wideband underwater acoustic devices, which will enable various applications in the future.

## Introduction

The stealth of low-frequency acoustics is challenging, particularly for underwater acoustics, because it has a longer wavelength than air sound. The internal friction in the linear systems of subwavelength thickness materials is very small; thus, the performance of conventional acoustic absorbing materials is poor in absorbing low-frequency underwater acoustic waves (specifically below 1 kHz). The rapid development of metasurface^1,2^ has open a novel pathway to control the electromagnetic waves in various applications, such as invisible cloak^3–5^, antenna^6–8^, beam control^9–11^ and so on. The similar concept is extended to the acoustic for the regulation of acoustic waves^12,13^, resulting in many novel acoustic devices such as acoustic vortices^14,15^, non-diffraction beams^16,17^, diffuse reflection^18,19^, and acoustic absorption^20–24^. However, majority of acoustic metasurfaces have focused on aeroacoustics, which is simpler because hard materials are applied in the meta-structure design. For underwater acoustics, the design and selection of material parameters are more complex because of the minor impedance mismatch between water and normal hard materials, such as metals. To date, there have been some proposals regarding underwater acoustic absorption^25–27^; for instance, using a design principle that maximised the thermoviscous loss, a matasurface for underwater sound absorption is proposed^26^. The experimental results indicate that multitudes of absorption peaks are generated in the range between 2 and 5 MHz. However, the existing work on low-frequency band performance is poor, and the design of the structure cannot achieve the broadband work effect.

The main reason for the difficulty in achieving broadband low-frequency underwater acoustic absorption is that it is very difficult to design and optimise the structural parameters of broadband response metasurfaces. Recently, the emergence of artificial intelligence has provided considerable convenience for the design and optimisation of metasurfaces. Utilising the advantage of self-learning of artificial intelligence, many achievements have been made in the field of reverse problem in metamaterials and metasurfaces^28–34^. For instance, combining forward modelling and inverse design in a tandem architecture, a novel inverse design method of photonic devices is proposed^28^, which solved the non-uniqueness problem in training. Although significant progress has been made in the reverse design of metasurfaces in the field of electromagnetism, the design of low-frequency underwater acoustic metasurfaces remains elusive.

In this study, we propose a reverse design method for ultra-thin low-frequency reflective underwater-acoustic metasurfaces based on a tandem deep neural network (DNN). The DNN network consists of a pre-trained forward network (PNN) and a reverse network (RNN) in series. A low-frequency broadband ultra-thin scattering underwater acoustic metasurface is proposed. An underwater broadband stealth effect was achieved by arranging the elements using pseudorandom method. In the frequency band between 700 and 1200 Hz, the energy loss of the metasurface in the echo direction was greater than 10 dB. This may be a novel way for achieving low-frequency broadband underwater acoustic stealth.

## Results

### Reverse design method

**Figure 1:**
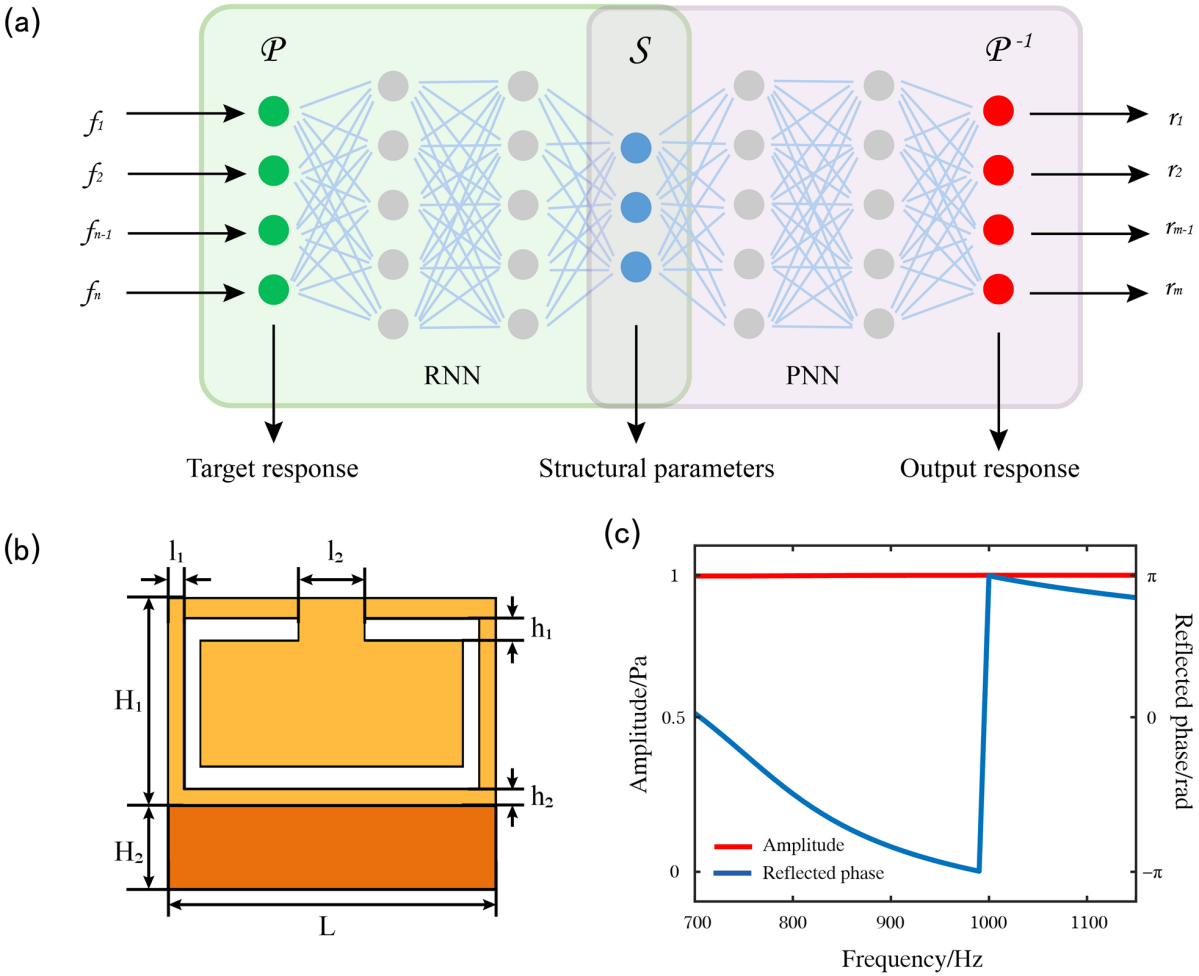
Reverse design method network structure and numerical simulation of a unit cell. (**a**) The schematic diagram of the reverse design method network structure, consisting of an RNN and a PNN. (**b**) The unit structure designed. It consists of ceramic resin with high toughness and high temperature resistance and air cavity. There are four structural variables in the air cavity, h_1_, h_2_, l_1_, l_2_. l_1_ represents the size of notch that satisfies l_1_ = L − 2l_2_; h_1_, h_2_, and l_2_ represent the size of air cavity. (**c**) The amplitude and phase of reflection response. The blue and red curves represent the amplitude and phase, respectively.

One major problem of the DNN during training is the non-uniqueness problem in reverse design, which means that different structural parameters can produce the same acoustic reflection response. To solve this problem, we display a tandem DNN cascaded by an RNN and a PNN, as shown in Fig. 1(a). The input layer is the target reflection response which consists of a 1 × 46 one-dimensional linear tensor, intermediate layer is the structural parameters which consists of a 1 × 4 one-dimensional linear tensor, and output layer is the predicted reflection response, which consists of a 1 × 92 one-dimensional linear tensor that is in serial output by the 46 real-part values and 46 imaginary-part values. During the training of the tandem DNN, we pretrained the PNN and fixed its network parameters, which were connected in series after the RNN, and only the RNN parameters were updated to reduce the loss function. Therefore, the designed structural parameters of the elements can be extracted from the intermediate layer.

The structure to be designed is shown in Fig. 1(b), and it is composed of a ceramic resin with high toughness and high-temperature resistance. There is a notched air cavity inside. The width, L, and thickness, H_1_, of the element structure are 10 and 5 cm, respectively; h_1_, h_2_, l_1_, and l_2_, which are the size of the cavity, are design variables. A steel plate with H_2_ of 3 cm and L of 10 cm was placed at the bottom of the element to enhance the underwater compression resistance. The reflection responses of interest are set in the low frequency underwater acoustic segment from 700 to 1150 Hz (1.27 m to 2.08 m in underwater acoustic wavelength, the H_1_ of the element is only 1/25 of the operating wavelength) for demonstration.

The corresponding reflection amplitude and phase of the element were simulated using the commercial finite-element package, COMSOL Multiphysics, as illustrated in Fig. 1(c). The blue and red curves represent the reflected amplitude and phase, respectively. The acoustic-solid coupling module was used for the simulation. The simulation boundary was periodic. The upper part of the element is the water area, whose acoustic velocity and density are 1460 m/s and 1000 kg/m^3^, respectively, and the pressure acoustic module is used for the simulation. The lower part of the element and the air cavity in the element are the air area, whose acoustic velocity and density are 343 m/s and 1.21 kg/m^3^, respectively, and the pressure acoustic module is also used for the simulation. The ceramic resin with high toughness and high-temperature resistance of the element is simulated by a solid mechanics module, whose Young's modulus, density, and Poisson's ratio are 10.5 GPa, 1610 kg/m^3^, and 0.32, respectively. The metal of the element was also simulated by a solid mechanics module, whose Young's modulus, density, and Poisson's ratio are 216 GPa, 7800 kg/m^3^, and 0.3, respectively. The reflection amplitude remained relatively stable without considering the material loss. We focused on the inverse design of the reflection response and element structure. To avoid periodic oscillation of the phase, the real and imaginary parts are used to uniquely determine the phase in the range of −π–π. Therefore, we selected the real and imaginary parts of the reflected response as the training dataset for the PNN. This significantly improves the prediction accuracy and efficiency of the PNN.

**Figure 2:**
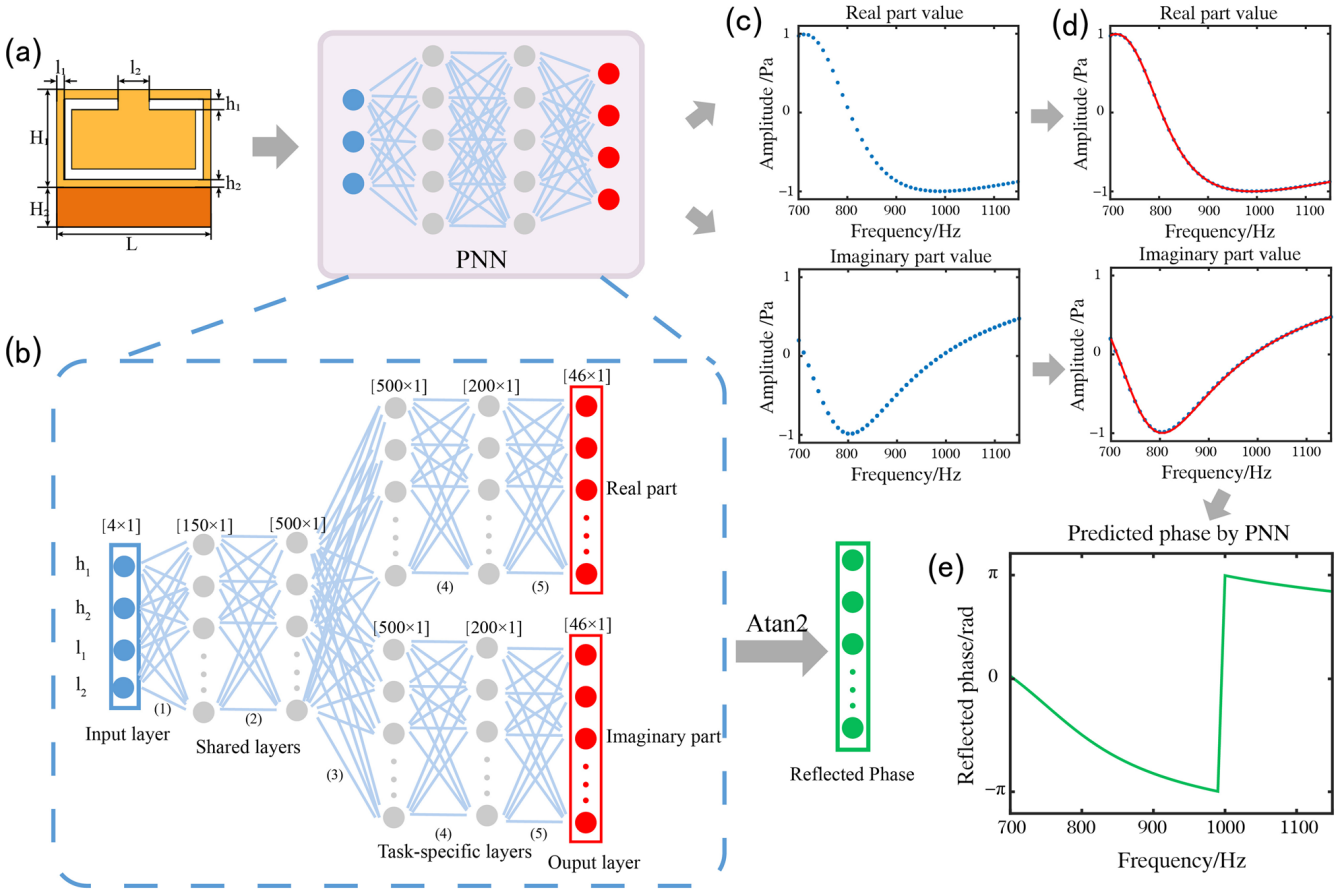
Pre-trained forward predictive design method. (**a**) Input of the PNN, which includes four structural parameters. (**b**) PNN, which is a multi-task DNN with two shared layers and three task-specific layers. The PNN can predict the real and imaginary parts values of reflection response independently. The blue, gray, and red circles represent the input layer, hidden layers and output layer of the PNN, respectively. (**c**) The real and imaginary part discrete values given by the PNN. (**d**) Continuous results obtained using the interpolating method. The blue curve and red point curve represent the continuous results and discrete values, respectively. (**e**) The corresponding reflected phase.

Before training the tandem network, the forward neural network, i.e., PNN, must be trained in advance; because of the one-to-one correspondence between the input and output. The concrete structure of the proposed PNN is illustrated in Fig. 2(b). It addresses the regression problem between the internal structural parameters and the reflection responses of underwater acoustic metasurface elements. The PNN is a multitask fully connected network that trains the real and imaginary parts separately. The input layer has four neurones, which represent four structural parameters (Fig. 2a). The hidden layer consisted of two shared layers and two task-specific layers. The former has 150 and 500 neurones, and the latter has 500 and 200 neurones. The output layer has an independent dual-task layer, and each task has 46 neurones, which represent the real and imaginary part values of the reflection responses, as illustrated in Fig. 2(c). The continuous value of the PNN output in the frequency range between 700 and 1150 Hz was obtained using the interpolating method (Fig. 2d). Using the inverse trigonometric function, the phase of the reflection response of the PNN output can be calculated using the following equation (Fig. 2e):

1 $$Phase=arctan2 \frac{Imag({S}_{11})}{Real({S}_{11})}$$

Using the commercial finite-element package, COMSOL Multiphysics, and MATLAB to co-simulate, 111, 200 groups of data were collected, of which 80%, 10%, and 10% were used for the training, validation, and test datasets, respectively. The ranges of the four variables in the order of h_1_, h_2_, l_1_, and l_2_ are [1–5 mm], [1–10 mm], [10–90 mm], and [1–10 mm], respectively. The four variables were normalised before inputting into the PNN to eliminate the influence of different orders of magnitude on the network prediction. The normalised variables were encapsulated into a 1 × 4 one-dimensional linear tensor and fed into the PNN. The output is a 1 × 92 one-dimensional linear tensor, which is in serial output by the 46 real-part values and 46 imaginary-part values. L2 was used as the loss function for the PNN, known as the mean square error (MSE). For a multitask network, the loss functions for the gradient descent method to update the network parameters are represented by the following equations:

2 $${L}_{real}= \frac{1}{N}\sum_{i=1}^{N}{({Real}_{prediction}-{Real}_{simulation})}^{2}$$

3 $${L}_{imag}= \frac{1}{N}\sum_{i=1}^{N}{({Imag}_{prediction}-{Imag}_{simulation})}^{2}$$

and

4 $${Loss}_{PNN}= \frac{1}{2}({L}_{real} + {L}_{imag})$$

**Figure 3:**
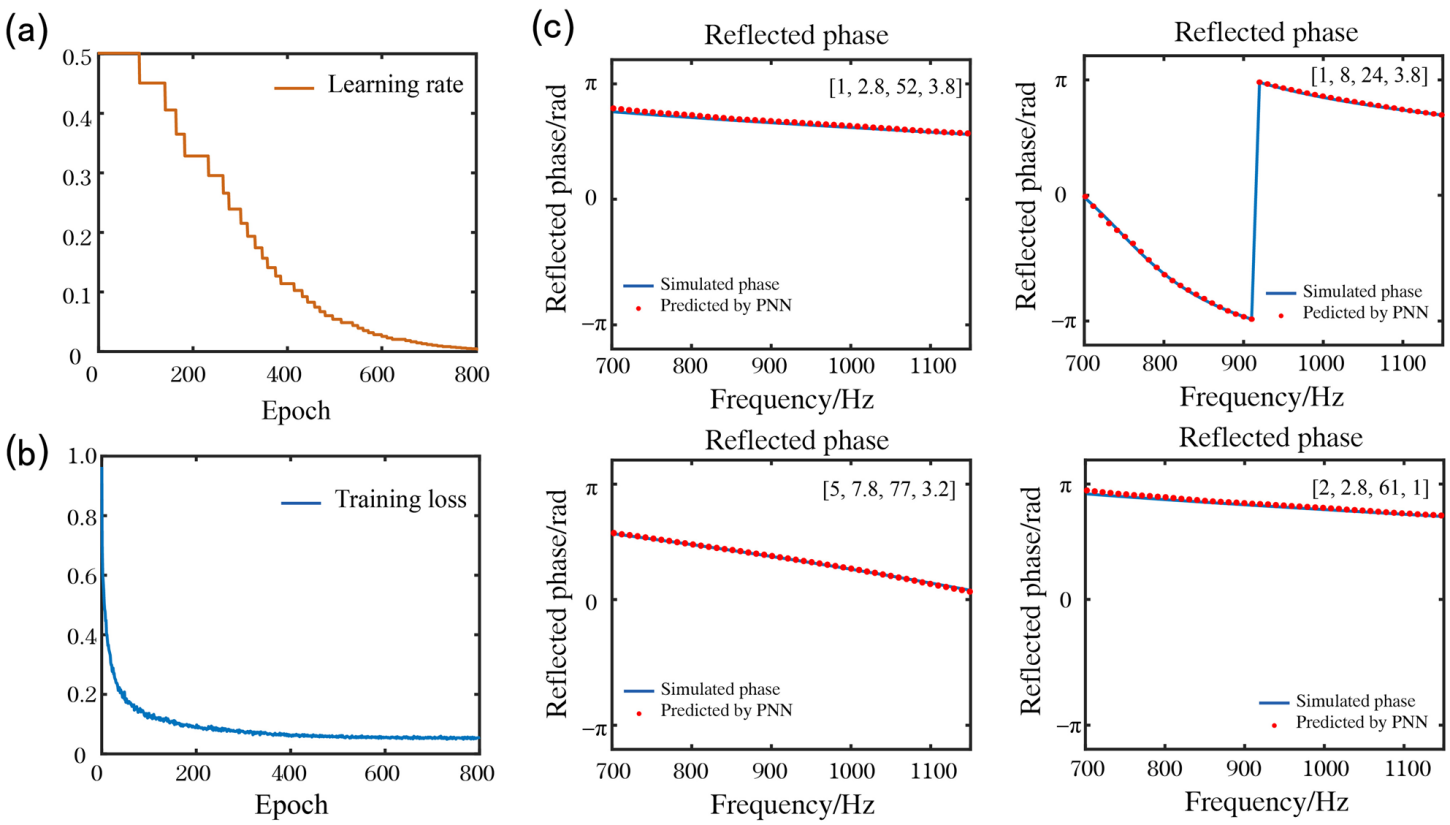
Loss and test samples of the PNN. (**a**) Dynamic learning rate. (**b**) The loss curve during PNN training. (**c**) Four examples of the PNN network prediction. The red point curves represent the reflected phase predicted by the PNN. The blue curves represent the reflected phase obtained from numerical simulations. The structural parameters of each unit cells are given in the inset in the following order: h_1_ (mm), h_2_ (mm), l_1_ (mm), and l_2_ (mm). All four elements presented are randomly selected from the test data.

The stochastic gradient descent method was adopted for the PNN training. The learning rate was dynamically adjusted according to the decrease in loss during training. The initial learning rate was set as 0.5. Figure 3(a) and (b) show the curves of changes in the learning rate of the network and the loss function changes of the training datasets during training. After the training was completed, the MSE for the test dataset was 0.0075. Several prediction examples randomly selected from the test dataset are shown in Fig. 3(c), and their structural parameters are shown in the inset. These examples demonstrate an outstanding consistency between the output (blue curve) of the PNN and the numerical simulation results (red point curve).

**Figure 4:**
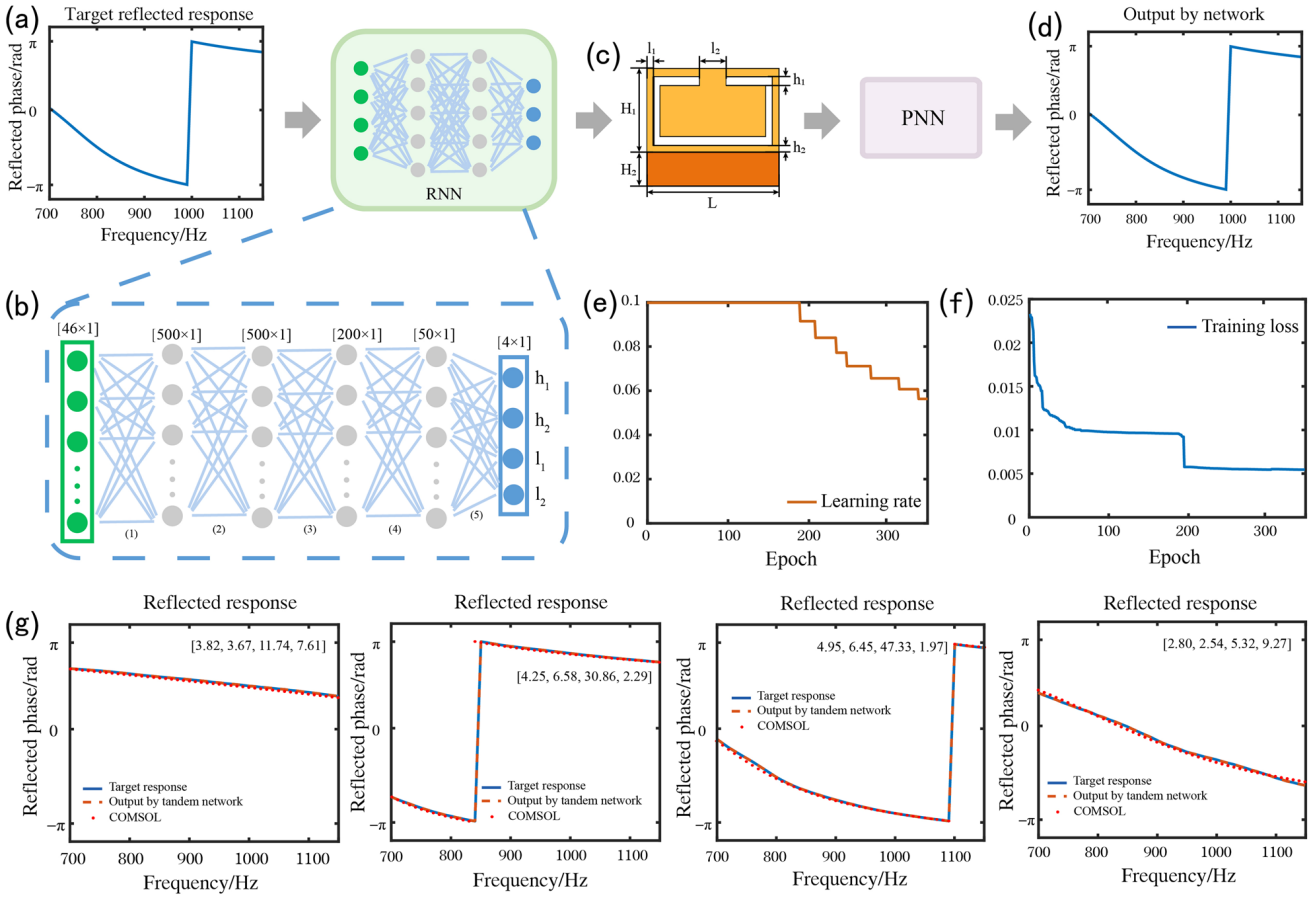
Reverse design method. (**a**) A target reflection phase curve. (**b**) RNN, which consists of a five-layer fully connected DNN. (**c**) Output of the RNN, which is a combination of the design parameters. (**d**) The designed reflection phase curve output by the PNN. (**e**) Dynamic learning rate. (**f**) The loss curve during the RNN training. (**g**) Several design samples from the proposed RNN on reflected phase design targets. Blue curves represent the target filter spectral responses. Brown dotted curves represent the PNN predicted reflection responses based on the designs given by the RNN. Red point curves represent the numerical simulation results of reflection responses based on the designed structural parameters. All designed parameters including h_1_ (mm), h_2_ (mm), l_1_ (mm), and l_2_ (mm) are given as insets.

With the successful training of the PNN, we connected it to the RNN to form a complete tandem network. The detailed architecture of the RNN is illustrated in Fig. 4(b). An RNN consists of a five-layer fully connected network, whose input and output layers have 46 and 4 neurones, respectively. The hidden layers have500, 500, 200, and 50 neurones. The RNN input was the target reflection response (Fig. 4a). The output of the RNN is the designed structural parameter of the element after normalisation. The real element structural parameters can be obtained by inverse normalisation (Fig. 4c). To eliminate the multi-solution problem, the RNN connects the PNN with parameter freezing in series. Only the parameters of the RNN are updated to reduce its loss function, which is the difference between the target reflection response and the prediction response (Fig. 4d), which is expressed as follows:

5 $${Loss}_{RNN}=\frac{1}{N}\sum_{i=1}^{N}{({Phase}_{target}-{Phase}_{prediction})}^{2}$$

An adaptive moment estimation optimiser was used for the RNN training. The training, validation, and test datasets selected by the RNN during training are the same as those of the PNN. The learning rate is also dynamically adjusted in the same manner as the PNN according to the decrease in loss during training. The original learning rate was set to 0.01. After training, the MSE of the test dataset loss was 0.01. Because the randomly generated target reflection response may be physically unrealistic, the stabilised error values indicate that training has been completed. The training loss and learning rate are shown in Fig. 4(e) and (f), respectively. Several design samples from the proposed RNN for the reflected phase design targets are shown in Fig. 4(g). The designed structural parameters after inverse normalisation are given as insets. Excellent consistency exists between the target reflection response (blue curve), network prediction (brown dotted curve), and numerical simulation results (red curve).

### Reverse design of elements

Based on the perfective tandem network, we designed a group of elements with a π/4 phase-shift interval in a wide band. We selected a reflection response from the test dataset as the first target reflection response, which was highly consistent with the network prediction and numerical simulation results. The other target reflection responses were transformed from the first target reflection response. Based on the first target reflection response, keeping the curve slope unchanged, a group of target reflection responses with adjacent phase intervals of π/4 can be obtained by shifting π/4 successively.

**Figure 5:**
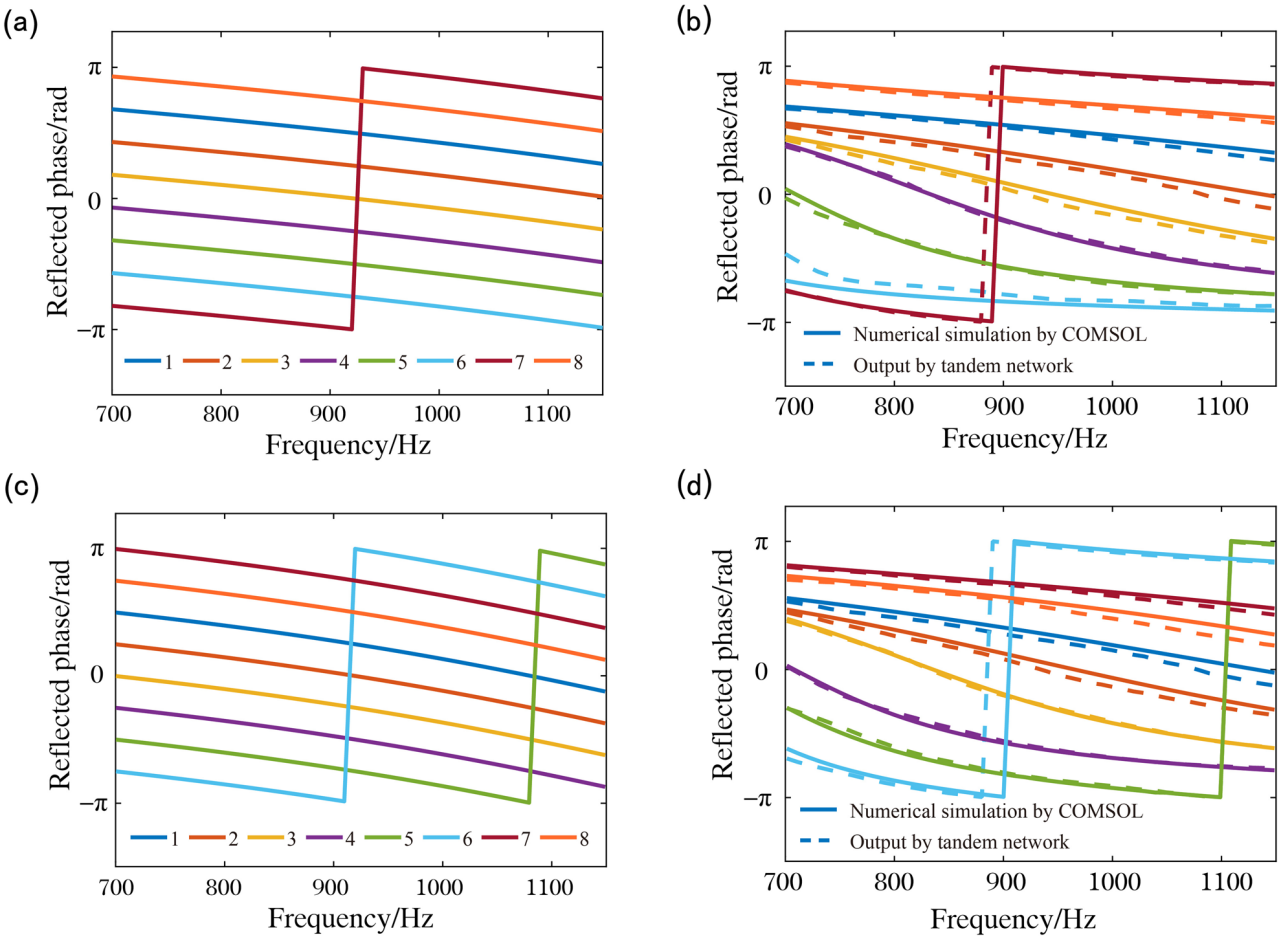
Reverse design of unit cells with π/4 phase shift interval. (**a**) A set of target reflection phases. (**b**) Comparison of the reflection phase for network prediction and simulation. The solid and dotted curves represent the prediction results by the tandem network and the simulation results by COMSOL, respectively (**c**, **d**). Another example of reverse design of underwater acoustic metasurface with the π/4 phase shift interval.

We designed two different target reflection phases, as illustrated in Fig. 5(a) and (c). By inserting the two designed groups of target reflection responses into the tandem network, we obtained the normalised element structural parameters by extracting the intermediate layer. Realistic designed structural parameters of the two different target reflection responses after inverse normalisation are provided in the Supplementary Materials. The predictive outputs of the tandem network are shown in Fig. 5(b) and (d). Comparing the target reflection responses and the prediction outputs, some of them agree well, while others do not a good match. Because the generated target reflection responses may be physically unrealistic, the tandem network can only predict the reflection response closest to the target reflection response based on physical reality.

We simulated the reflection responses with the structural parameters predicted by the reflection responses of the two groups of target reflection responses. The numerical simulation results of the elements were obtained using the commercial finite element package, COMSOL Multiphysics. Comparing the prediction outputs and the numerical simulation results, the prediction outputs of the real and imaginary parts are slightly different from the numerical simulation results, and the reflected phases are highly consistent.

### Design and simulation of diffuse underwater acoustic metasurface

**Figure 6:**
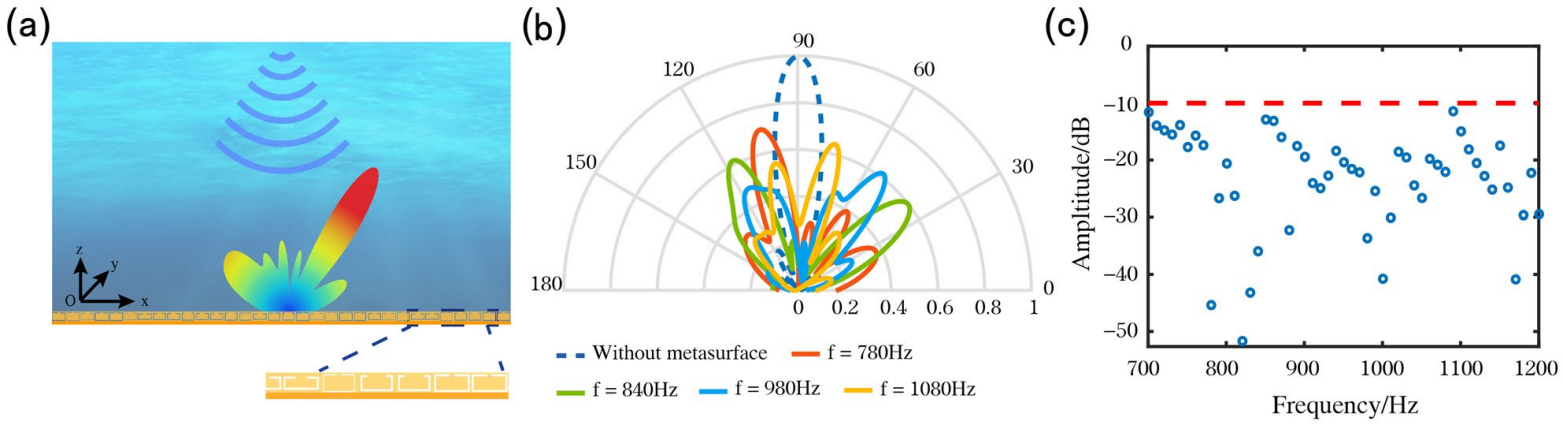
Design of wide-band diffuse underwater acoustic metasurface. (**a**) Schematic diagram of wide-band diffuse underwater acoustic metasurface. (**b**) Simulation results of reflection acoustic far field at 780 Hz, 840 Hz, 980 Hz and 1080 Hz, respectively. The solid and dotted curves represent the acoustic far field with or without metasurface, respectively. (**c**) Energy loss in the direction of the echo in the frequency band between 700 and 1200 Hz.

Based on the inverse design of the two groups of target elements, we utilise example two to realize a low-frequency and wideband diffuse underwater-acoustic metasurface. During the actual design, we made an entire diffuse underwater-acoustic metasurface plane a flat rectangle with a side length of 600 mm. A general view of the plane is displayed in Fig. 6(a), with the x direction comprising 60 elements and the y direction having infinite length. The arrangement of the metasurface was generated using the pseudorandom method in the x-direction.

Figure 6(b) illustrates the diffuse acoustic far-field contrast of with or without diffuse underwater-acoustic metasurface after normalization at different frequencies. By contrast, the main lobe of acoustic far-field energy without metasurface is concentrated in the echo direction, and the secondary lobe energy is very low, with almost no energy scattering in other directions. Most of the energy of the acoustic far-field with metasurface is scattered in other directions, without almost little energy in its echo direction. Figure 6(c) demonstrates the simulation results in the frequency range between 700 and 1200 Hz. The results indicate that the echo energy loss of the proposed diffuse acoustic metasurface is greater than 10 dB in the frequency band ranging from 700 to 1200 Hz, indicating that it scatters most of the energy in other directions in this frequency band, which can achieve the diffuse reflection effect in the low and wide frequency band.

## Conclusion

In conclusion, we propose a novel tandem network approach to reverse the design of acoustic elements. Based on this method, we designed a set of underwater acoustic metasurface elements with flat phase changes and nearly equal phase-shift intervals in 700–1150 Hz. A diffuse underwater acoustic metasurface composed of 60 elements was designed using the designed metasurface elements. Simulation results indicate that the diffuse reflection effect of the broadband can be realised in this frequency band by arranging the elements using pseudorandom method, and the energy loss in the echo direction can exceed 10 dB. Our work opens a novel pathway for realising low-frequency wideband underwater acoustic devices, which will enable various applications in the future.

## Supplementary Information

Supplementary Information.

## Data Availability

The executable codes and datasets are available from the corresponding author on reasonable request.
