# Supplementary material for: Design of ultra-thin underwater acoustic metasurface for broadband low-frequency diffuse reflection by deep neural networks
Source: Sci Rep. 2022 Jul 14;12:12037. doi: 10.1038/s41598-022-16312-1 (PMC9283483; doi:10.1038/s41598-022-16312-1)
Supplement: Supplementary file 1 — Supplementary Information. [file 41598_2022_16312_MOESM1_ESM.pdf]

Supplementary Materials

Design of ultra-thin underwater acoustic metasurface for broadband low-frequency diffuse reflection by deep neural networks

Ruichen Li[1], Yutong Jiang[1], Rongrong Zhu[1, 2, *], Yijun Zou[1], Lian Shen[1], and Bin Zheng[1, 3, 4, *]

[1] *Interdisciplinary Center for Quantum Information, State Key Laboratory of Modern Optical Instrumentation, ZJU-Hangzhou Global Scientific and Technological Innovation Center, Zhejiang University, Hangzhou 310027, China.*

[2] *School of Information and Electrical Engineering, Zhejiang University City College, Zhejiang, 310015, China*

[3] *International Joint Innovation Center, Key Lab. of Advanced Micro/Nano Electronic Devices & Smart Systems of Zhejiang, The Electromagnetics Academy at Zhejiang University, Zhejiang University, Haining 314400, China*

[4] *Jinhua Institute of Zhejiang University, Zhejiang University, Jinhua 321099, China*

*Correspondence: Rongrong Zhu (rorozhu@zju.edu.cn), Bin Zheng (zhengbin@zju.edu.cn)

Two sets of target reflection responses are selected to verify the performance of the network. The structural parameters of elements reversely designed by the network are output from the intermediate layer of the network, showing in Tables S1 and S2.

Table. S1. The designed structural parameters of example one

Number	h_1[mm]	h_2[mm]	l_1[mm]	l_2[mm]
1	3.30	3.83	12.83	6.58
2	1.00	3.00	10.36	10.00
3	1.00	2.58	10.09	10.00
4	5.00	2.55	10.02	5.67
5	5.00	2.73	10.00	1.27
6	5.00	2.23	10.00	1.00
7	4.98	4.33	59.25	1.56
8	4.88	5.87	20.30	1.01

Table. S2. The designed structural parameters of example two

Number	h_1[mm]	h_2[mm]	l_1[mm]	l_2[mm]
1	1.02	2.99	10.40	9.99
2	1.00	2.62	10.19	10.00
3	5.00	2.50	10.04	6.07
4	5.00	2.65	10.00	1.75
5	4.99	3.89	70.63	1.51
6	4.80	6.09	41.31	1.85
7	4.98	5.38	15.37	1.00
8	4.60	4.84	11.06	1.18
